# Eradication of *Helicobacter pylori* Is Associated with the Progression of Dementia: A Population-Based Study

**DOI:** 10.1155/2013/175729

**Published:** 2013-11-25

**Authors:** Yang-Pei Chang, Guei-Fen Chiu, Fu-Chen Kuo, Chiou-Lian Lai, Yuan-Han Yang, Huang-Ming Hu, Pi-Yu Chang, Chiao-Yun Chen, Deng-Chyang Wu, Fang-Jung Yu

**Affiliations:** ^1^Department of Neurology, Kaohsiung Medical University Hospital, Kaohsiung 807, Taiwan; ^2^Department of Information, Kaohsiung Municipal Ta-Tung Hospital, Kaohsiung 801, Taiwan; ^3^School of Medicine, College of Medicine, I-Shou University and E-Da Hospital, Kaohsiung 824, Taiwan; ^4^Graduate Institute of Public Health, Kaohsiung Medical University, Kaohsiung 807, Taiwan; ^5^Division of Gastroenterology, Department of Internal Medicine, Kaohsiung Medical University Hospital, Kaohsiung 807, Taiwan; ^6^Department of Medicine, Faculty of Medicine, College of Medicine, Kaohsiung Medical University, Kaohsiung 807, Taiwan; ^7^Administration Center, Kaohsiung Medical University Hospital, Kaohsiung 807, Taiwan; ^8^Department of Radiology, Faculty of Medicine, College of Medicine, Kaohsiung Medical University, Kaohsiung 807, Taiwan; ^9^Cancer Center, Kaohsiung Medical University Hospital, Kaohsiung 807, Taiwan; ^10^Department of Internal Medicine, Kaohsiung Municipal Hsiao-Kang Hospital, Kaohsiung 812, Taiwan

## Abstract

*Objective*. To evaluate the effect of eradication of *Helicobacter pylori* (*H. pylori*) on the progression of dementia in Alzheimer's disease (AD) patients with peptic ulcer. *Methods*. Participants with the diagnosis of AD and peptic ulcer were recruited between 2001 and 2008. We examined the association between eradication of *H. pylori* and the progression of AD using the multiple regression models. Medication shift from Donepezil, Rivastgmine, and Galantamine to Mematine is defined as progression of dementia according to the insurance of National Health Insurance (NHI) under expert review. *Results*. Among the 30142 AD patients with peptic ulcers, the ratio of medication shift in AD patients with peptic ulcers is 79.95%. There were significant lower incidence comorbidities (diabetes mellitus, hypertension, cerebrovascular disease, coronary artery disease, congestive heart failure and hyperlipidemia) in patients with *H. pylori* eradication as compared with no *H. pylori* eradication. Eradication of *H. pylori* was associated with a decreased risk of AD progression (odds ratio [OR] 0.35 [0.23–0.52]) as compared with no *H. pylori* eradication, which was not modified by comorbidities. *Conclusions*. Eradication of *H. pylori* was associated with a decreased progression of dementia as compared to no eradication of *H. pylori* in AD patients with peptic ulcers.

## 1. Introduction

Alzheimer's disease (AD) is a common neurodegenerative disorder for which causes are diverse, and it involves similar neuroinflammation cascade as prion disease [1]. Cerebral amyloid deposits are colocalized with a broad variety of inflammation-related proteins (complement factors, acute-phase protein, and proinflammatory cytokines) and clusters of activated microglia [2]. Currently, identified risk factors of AD include age, sex, plasma homocysteine level, and genetic factors like apolipoprotein E allele *ε*4 [3, 4]. Several studies have shown the association between infection and AD, including HSV-1, *Chlamydia pneumonia*, spirochetes, and *Helicobacter pylori* (*H. pylori*) [5–8]. As for *H. pylori* infection, previous case-control studies found an association between *H. pylori* and AD. An impressive intervention study has shown positive results that the *H. pylori* eradication may improve the cognitive functiona outcome within two years, but the sample size of case (28 patients) and controls (16 patients) might be small for application to general population [9]. Additionally, some agents like statins, inhibitors of 3-hydroxy-3-methylglutaryl coenzyme A (HMG-CoA) reductase, have also shown to potentially attenuate neuroinflammatory processes and play a role in halting the degeneration process of AD [10–13]. A recent case-control study also showed preliminary results that AD patients with *H. pylori* infection may be more cognitively impaired. Roubaud-Baudron et al. found higher CSF cytokine (TNF-*α*, IL-8) and significantly positive correlation between *H. pylori* immunoglobulin level and homocysteine level and they concluded that the impact of *H. pylori* infection on AD course may be attributed to cerebrovascular lesions and neuroinflammation [14]. These observations led to the hypothesis that eradication of *H. pylori* infection modulating neuroinflammatory process may have a protective role for AD. Given that Taiwan's National Health Insurance Reimbursement Policy requested physicians to perform eradication of *H. pylori* infection depending on gastrointestinal endoscopy biopsy with or without 13C-urea breath test and neurologists to treat worsening of AD patients with anti-cholinesterase treatment according to repeated neuropsychological assessment and detailed expert review, we were allowed to observe the impact of eradication of *H. pylori* on the AD course.

## 2. Methods 

### 2.1. Data Source

The database used in this study included one million randomly selected subjects from the 1996–2007 Taiwan National Health Insurance Research Database (NHIRD), which was developed for research purposes. The NHIRD is a research database developed at the National Health Research Institute, with linked data from the demographic and enrollment records, hospital claims, ambulatory care visits, and pharmacy dispensing claims from hospitals, outpatient clinics, and community pharmacies. Our source population comprised all beneficiaries from the Longitudinal Health Insurance Database 2005 who were at least 50 years of age on January 1, 2001. There were no statistically significant differences in age, gender, or average insured payroll-related amount between the sample group and all enrollees.

*Study Population.* From the NHID, AD patients who were collected from outpatient pharmacy database between January 1, 1997, and December 31, 2004, with a primary diagnosis of dementia (Classifications of Diseases-9 codes: 290.xx) and regularly taking anticholinesterase medications (include donepezil, rivasitgmine, or galantamine according to anatomical therapeutic chemical (ATC) classification system codes provided in Supplemental Table 1 in the supplementary material available online at http://dx.doi.org/10.1155/2013/175729) for more than 3 months. We then selected AD patients with the diagnosis of peptic ulcer (Classifications of Diseases-9 codes: 531–534, A-code: A534). Patients who received *H. pylori* eradication therapy and those who did not receive *H. pylori* eradication therapy were classified into two subgroups. Due to Taiwan's National Health Insurance Reimbursement Policy request, worsening of neuropsychological assessment including minimental status exam may shift anticholinesterase medications (donepezil, rivastgmine, or galantamine) to memantine and defined as worsening of dementia; AD patients who shift or did not shift anti-cholinesterase medications were analyzed separately. Comorbidities were defined as diseases diagnosed before the index outpatient clinic visit.

### 2.2. *H. pylori* Eradication Method

*H. pylori* eradication with triple or quadruple therapy was defined as proton pump inhibitor or H2 receptor blocker, plus clarithromycin or metronidazole, plus amoxicillin or tetracycline, with or without Bismuth (details for all eligible *H. pylori* eradication regimens are reported previously) [15]. These drug combinations were prescribed within the same prescription order, and the duration of therapy was between 7 and 14 days. One year was chosen as the cutoff value based on the distribution of *H. pylori* eradication date after index hospitalization.

### 2.3. Covariate Ascertainment and Adjustment

We used inpatient and outpatient diagnosis files and prescription files during the 12-month period before the index date to ascertain patients' history of hypertension diabetes mellitus, cerebrovascular disease, coronary artery disease, congestive heart failure, and hyperlipidemia (ICD-9-CM codes provided in Supplemental Table 2); we also collected patient information on age, sex, and resource utilization (number of outpatient visits, number of hospitalizations, number of laboratory test measurements) 12 months prior to the index date.

### 2.4. Statistical Analysis

Baseline characteristics, comorbidities, and medication use were presented. For all cohort members, we computed their person days of followup in each anti-cholinesterase medication category. We examined the effect of *H. pylori* eradication therapy on risk of shifting anticholinesterase medications by comparing the occurrence of shifting medications after the *H. pylori* eradication therapy among AD patients with peptic ulcers. Multiple regression model was used to calculate the odds ratios (ORs) and their 95% CIs.

## 3. Results

**Figure 1:**
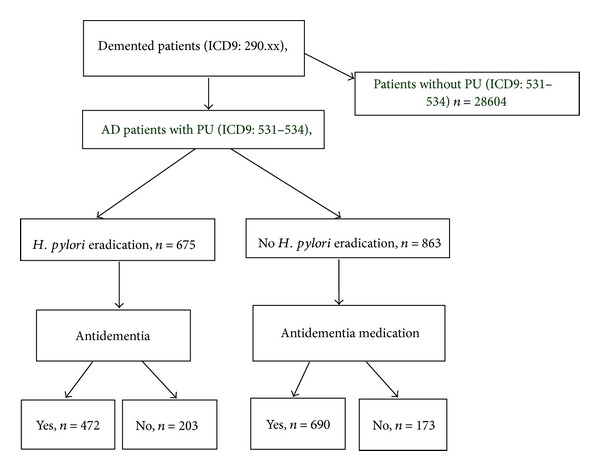
Flow chart of the study cohort assembly from prescriptions in Taiwan's National Health Insurance Research Database. Taiwan National Health Insurance Database, 1 million subtract from 23 million.

After excluding subjects who did not meet our study criteria, a total of 30142 AD patients were included in the analysis (Figure 1). A total of 1538 AD patients with peptic ulcer were then selected and classified into two groups: with *H. pylori* eradication (*n* = 675) and without *H. pylori* eradication (*n* = 863). Among these two groups enrolled in our study, several baseline characteristics, including diabetes mellitus, hypertension, cerebrovascular disease, congestive heart failure, coronary artery disease, and hyperlipidemia, were higher in frequency in patients without *H. pylori* eradication (Table 1). According to our previous study, the eradication rate of *H. pylori* was 89.4% to 90.5% [16]. Thus, it was estimated that 90% of our patients (*n* = 675) had successful eradication of *H. pylori*. Our results would underestimate the association between eradication therapy of *H. pylori* and the progression of AD.

As compared with no *H. pylori* eradication, *H. pylori* eradication was associated with a decreased risk of changing anticholinesterase medication with the OR of 0.58 (95% CI 0.46–0.74). The protective association of *H. pylori* eradication persisted with OR of 0.34 (95% CI 0.23–0.52) after adjustment of comorbidities (Table 2).

## 4. Discussion

Our study demonstrated that eradication therapy of *H. pylori* has a decreased association with AD progression as compared to no *H. pylori* eradication in AD patients with peptic ulcer, after adjusting for age, sex, comorbidities, and other potential confounder medications. We also observed a higher frequency of comorbidities in patients without eradication therapy of *H. pylori*. Previous reports also suggested the association between *H. pylori* infection and diabetes mellitus, hypertension, and cerebrovascular disease [17, 18]. In patients with diabetes mellitus, *H. pylori* infection or seropositivity not only increases the risk of atherosclerosis and cardiovascular disease but also contributed to promoting insulin resistance and increased microalbuminemia [18, 19].

Chronic *H. pylori* infection has shown to increase gastric pH level of gastric juice and thus leads to reduced folate absorption and increased blood homocysteine level, both of which would result in the damage of vascular endothelial cells and increased the risk of atherosclerosis [20–23]. However, the link between cerebrovascular disease and *H. pylori* infection still needs prospective studies [14, 24]. Our study may provide some support for the hypothesis of *H. pylori* infection causing atherosclerosis and risk of cerebrovascular disease.

Indeed, vascular risk factors, including adult-onset diabetes mellitus, hypertension, atherosclerosis disease and atrial fibrillation, are known to increase the risk of AD [25]. Cerebral vascular dysfunction may cause the accumulation of amyloid-beta (Abeta) protein and neuroinflammation in animal model, and reduction of fibrillar Abeta protein deposition may ameliorate the neuroinflammation [26, 27]. Neuroinflammation disrupting the blood brain barrier, together with fibrinogen, may be one of the contributing factors for familial cerebral amyloid angiopathy and Alzheimer's disease [28, 29]. It is plausible to propose that antibacterial treatment of chronic inflammation caused by *H. pylori* or other pathogens like spirochete may reduce neuroinflammation and thus prevent dementia [7].

The strength of our study is the enrollment of a nationally representative cohort of a large sample size. The information regarding anticholinesterase medications and eradication therapy of *H. pylori* is obtained by linking to the NHI pharmacy database under the Reimbursement Policy request of NHI to reduce the possibility of duplication or misclassification. Furthermore, covariates including underlying diseases (especially diabetes mellitus), were taken into consideration. However, there are several limitations. First, although we analyzed health care records from a national representative dataset of 1 million people, there were still few AD cases to allow us to have a precise estimation. Second, we did not adjust the use of medications that potentially may affect AD risk such as statins, NSAID, antidiabetic agents, calcium channel blockers, and neuroleptic agents. Third, we were not able to assess the genotype of apolipoprotein E allele *ε*4, which cement the solid relevance in late-onset AD [30–32]. Fourth, our diagnosis of AD was based on the diagnosis code from the NHI database; therefore, we were not able to distinguish between AD and mixed type dementia. Nonetheless, a stringent policy from NHI validates our diagnosis by expert review before the use of anticholinesterase medications. However, given that all the medical information from the NHI database was deidentified due to ethical privacy concern, we could not recognize all the AD-diagnosed subjects in our study and therefore did not have the opportunity to review all their medical charts. Last, we could not exclude the possibility that the observed association was due to sick-stopper effect (nonadherence to medication due to higher risk) or protopathic bias (less AD symptoms may increase the awareness of the importance of eradication therapy). Further longitudinal study including measures of neuropsychiatric assessment over time is needed to clarify the interrelated roles of cognition, eradication therapy of *H. pylori*, and AD.

## 5. Conclusion

We observed that eradication therapy of *H. pylori* had a deceased association with AD progression compared with no eradication therapy among AD patients with peptic ulcer. Further long-term follow-up study is needed to confirm the potential beneficial role of antibacterial therapy of *H. pylori* in AD.

## Supplementary Material

Table 1. Anatomical therapeutic chemical (ATC) for anticholinesterase medications.
Table 2. ICD codes for comorbid diseases.

## Figures and Tables

**Table 1:** Basic demographic data of demented patients with peptic ulcer.

	1538 demented patients with PU (ICD-9:290.xx & 531–534)		*P* value
*H. pylori* eradication	Yes, *n* = 675	No, *n* = 863	
Age, yr	79.41 ± 9.64	76.07 ± 11.59	
Female, %	338 (50.00)	475 (55.77)	0.0528
Diabetes mellitus, %	46 (6.81)	161 (18.65)	<0.0001
Hypertension, %	85 (12.59)	308 (35.56)	<0.0001
Cerebrovascular disease and TIA, %	57 (8.44)	277 (32.09)	<0.0001
Congestive heart failure, %	32 (4.74)	102 (11.81)	<0.0001
Coronary artery disease, %	42 (6.22)	185 (21.43)	<0.0001
Hyperlipidemia, %	45 (6.66)	159 (18.42)	<0.0001

PU: peptic ulcer. TIA: transient ischemic attack.

*P* value, by student's *t*-test.

**Table 2:** The effect of *H. pylori* eradication on the progression of dementia.

	1538 demented people with PU (ICD-9:290.xx & ICD-9:531–534)				
	*H. pylori* eradication		Percentage, %	OR, (95% CI)	Adjusted OR, (95% CI)
	Yes	No			
Medication change (+)	472	690	79.95	0.58 (0.46–0.74)	0.34 (0.23–0.52)
Medication change (−)	203	173	20.05	1	1

PU: peptic ulcer. OR: odds ratio. We estimated odds ratio of progression of dementia by *χ* ^2^ test.

We estimated adjusted odds ratio by multiple linear regression, adjusted with age, gender, diabetes mellitus, hypertension, cerebrovascular disease and TIA, congestive heart failure, coronary artery disease, and hyperlipidemia.
