# Supplementary material for: Eradication of Helicobacter pylori Is Associated with the Progression of Dementia: A Population-Based Study
Source: Gastroenterol Res Pract. 2013 Nov 25;2013:175729. doi: 10.1155/2013/175729 (PMC3859120; doi:10.1155/2013/175729)
Supplement: Supplementary file 1 — Table 1. Anatomical therapeutic chemical (ATC) for anticholinesterase medications. Table 2. ICD codes for comorbid diseases. [file 175729.f1.pdf]

Supplemental Table 1

ATC code	Name	DDD	U	Adm.R	Note
N06DA02	Donepezil	7.5	mg	O	
N06DA03	Rivastigmine	9	mg	O	
06DA04	Galatamine	16	mg	O	

Supplemental Table 2

Disease	ICD-9 CM
Hypertension	4010,4011,4019
Diabetes mellitus	25000,25002,20510,25012,25020,25022,25030,25032,25040,25042, 25050,25052,25060,25062,25070,25072,25080,25082,25090,25092
Cerebrovascular disease	24340,4341,4349,436
Coronary heart disease	4140,4141,4142,4243,4148,4149
Congestive heart failure	4280,4281,42820,42821,42822,42823,42830,42831,42832,42833, 42840,42841,42842,42843,4289
Hyperlipidemia	2720,2721,2722,2723,2724
